# Short-term efficacy of photobiomodulation in early and intermediate age-related macular degeneration: the PBM4AMD study

**DOI:** 10.1038/s41433-024-03326-4

**Published:** 2024-09-14

**Authors:** Marco Nassisi, Claudia Mainetti, Giorgia Rosapia Paparella, Luca Belloni Baroni, Paolo Milella, Gaia Leone, Davide Galli, Francesco Pozzo Giuffrida, Laura Dell’Arti, Chiara Mapelli, Giuseppe Casalino, Francesco Viola

**Affiliations:** 1 Ophthalmology Unit, Fondazione IRCCS Ca’ Granda Ospedale Maggiore Policlinico, Milan, Italy; 2 Department of Clinical Sciences and Community Health, University of Milan, Milan, Italy

**Keywords:** Retinal diseases, Therapeutics

## Abstract

**Objectives:**

This independent prospective study evaluated the short-term effects and safety of photobiomodulation (PBM) in early and intermediate age-related macular degeneration.

**Methods:**

patients were treated with PBM in one eye. Functional parameters and drusen volume were measured at one (W4), three- (W12) and six-months (W24) after PBM.

**Results:**

The study included 38 subjects who completed the PBM protocol. Two patients developed macular neovascularization during the study period. Best corrected visual acuity improved from 77.82 ± 5.83 ETDRS letters at baseline to 82.44 ± 5.67 at W12 (*p* < 0.01), then declined to 80.05 ± 5.79 at W24 (*p* < 0.01 vs. baseline). Low luminance visual acuity showed a similar pattern, improving from 61.18 ± 8.58 ETDRS letters at baseline to 66.33 ± 8.55 at W12 (*p* < 0.01), and decreasing to 62.05 ± 9.71 at W24 (*p* = 0.02). Contrast sensitivity improved at W12 (20.11 ± 9.23 ETDRS letters, *p* < 0.01), but returned to baseline by W24 (16.45 ± 9.12, *p* = 0.5). Scotopic microperimetry showed a decrease in mean absolute retinal sensitivity from 9.24 ± 3.44 dB to 7.47 ± 4.41 dB at W24 (*p* < 0.01), while relative sensitivity decreased only at W24 (*p* = 0.04). Drusen volume decreased at W4 (0.018 ± 0.009 mm3, *p* < 0.01) and W12 (0.017 ± 0.009 mm3, *p* < 0.01), with a slight increase at W24 (0.019 ± 0.012 mm3, *p* = 0.154).

**Conclusions:**

PBM resulted in temporary improvements in visual function and a reduction in drusen volume, but these effects were not sustained at six months. The long-term efficacy and impact on disease progression are uncertain, necessitating further research to confirm these findings and determine optimal patient selection.

## Introduction

Age-related macular degeneration (AMD) is a progressive and chronic retinal disease that primarily affects the macula, leading to irreversible central vision loss in the elderly population of developed countries. In 2020, AMD affected an estimated 196 million people worldwide [1–3]. The early and intermediate stages of AMD are characterized by the presence of drusen, while advanced stages involve macular neovascularization (MNV) and/or geographic atrophy (GA) [4, 5]. Current treatments target exudative MNV, and aim to slow the progression of atrophy in the absence of MNV [6, 7]. Unfortunately, no treatment options are currently available for the earlier stages of AMD [4, 8]. However, treating these earlier stages could potentially prevent or delay the irreversible central vision impairment observed in the late stages of the disease. Photobiomodulation (PBM) is a therapeutic approach that involves modulating cellular pathways using specific wavelengths of light that can be absorbed by photosensitive molecules [9]. Near-infrared (NIR) light, within the wavelength range of 500–1000 nm, is hypothesized to stimulate cytochrome C-oxidase in the mitochondria, leading to increased mitochondrial replication and density, enhanced cellular metabolic rate, and heightened antioxidant activity. PBM has demonstrated benefits in various diseases including orthodontic and dermatological conditions [10–15]. Although a few clinical trials have explored the use of PBM in early and intermediate AMD, with promising safety and visual outcome results, the efficacy and prognostic factors associated with PBM therapy remain to be determined [16–19]. In this independent, prospective, monocentric interventional cohort study, we aim to evaluate the short-term efficacy and safety of PBM in eyes with early or intermediate AMD and identify potential prognostic factors linked to treatment outcomes.

## Methods

The study was an independent, prospective, monocentric interventional cohort study. Consecutive patients with early or intermediate AMD were recruited at the Ophthalmology Unit of the IRCCS Ca’ Granda Foundation Ospedale Maggiore Policlinico, Milan, Italy, between January and April 2022. Eligible subjects had early or intermediate AMD [20], were aged ≥ 55 years, and had a best corrected visual acuity (BCVA) between 0 and 1 LogMAR.

Exclusion criteria included any form of late AMD in the study eye, pupillary abnormalities, active or previous sensitivity to yellow/red/near-infrared light, a history of or current treatment for epilepsy, and the presence of other ocular pathologies (e.g glaucoma, vitreopathies, myopic maculopathy, other retinopathies, advanced cataract) that could affect the results and treatment effectiveness. Only one eye per patient was treated with NIR-PBM. Notably, the presence of MNV and/or complete retinal pigment epithelium and outer retinal atrophy (cRORA) in the fellow eye was not an exclusion criterion.

The study protocol was approved by the local ethics committee (Comitato Etico Milano Area 2, protocol n.3136 of the 12^th^ of November 2021) and adhered to the tenets of the Declaration of Helsinki. All patients provided informed consent after the study and its potential outcomes were explained to them.

A blood test to assess levels of triglycerides and cholesterol (total, low-density lipoprotein [LDL] and high-density lipoprotein [HDL] cholesterol) was conducted at baseline.

### Ophthalmic assessment

A complete ophthalmic assessment of the study eye was performed during the screening visit and at 1 month (W4), 3 months (W12), and 6 months (W24) after the initial application (baseline).

All subjects were assessed for BCVA and low luminance visual acuity (LLVA) using ETDRS charts at a distance of 4 meters (Precision Vision, Woodstock, IL, USA). For LLVA, a 2.0 log unit neutral density filter was applied in front of the patient’s best correction [21]. Contrast sensitivity (CS) was also assessed using Sloan charts (Precision Vision, Woodstock, IL, USA). Retinal sensitivity was recorded in scotopic conditions using MP-1S (Nidek Technologies) with a personalized map of 60 tested points after a 30-minute dark adaptation period. Mean absolute retinal sensitivity was determined by averaging the results of all tested points, while mean relative retinal sensitivity was calculated by averaging the results of all tested points, excluding the point at 0 dB.

Subjects were assessed with 20° × 20° high speed SD-OCT volume scans (Spectralis OCT, Heidelberg Engineering, Heidelberg, Germany) consisting of 97 horizontal section scans (60 μm inter-scan distance, 30 frames averaged) and with 2 central (one vertical and one horizontal) 30° line scans, 30 times averaged in enhanced depth imaging (EDI) modality. Central retinal thickness (CRT) and drusen volume in the central millimeter (DV), 3 mm (DV3), and 6 mm (DV6) were automatically obtained with the machine’s software (Heidelberg Eye Explorer v1.10.4.0).

OCT images were also qualitatively assessed for the presence of subretinal drusenoid deposits (SDD), and hyperreflective retinal foci (HRF), as previously reported [22]. All qualitative and quantitative assessments were performed by two independent operators (LBB and GRP). In cases of disagreement, open adjudication was conducted between the two graders; unresolved cases were assessed by the head of the service (FV) who made the final decision.

Fundus autofluorescence (FAF) with a 488 nm wavelength (Spectralis OCT, Heidelberg Engineering, Heidelberg, Germany) was also performed at the same visit.

### Photobiomodulation

All included subjects were treated with PBM (Lumithera Valeda Light Delivery System, LumiThera Inc., Poulsbo, WA, USA) which delivers three different wavelengths in the NIR (850 nm), red (660 nm) and yellow (590 nm) ranges. Each session consists of four phases [17]: in the 1st and 3rd phases, the device delivers 35 s of pulsed yellow and NIR wavelengths while the patient’s eyes are open; in the 2nd and 4th phases, the device delivers 90 s of continuous red wavelength while the patient’s eyes are closed. Each session lasts a maximum of 5 min. Per the manufacturer’s instructions, all subjects received a total of 9 sessions (3 per week for 3 weeks, with a minimum of 24 h between consecutive applications). The first PBM application could be performed on the same day as the screening visit or within one week.

### Statistical analyses

IBM Statistical Package for Social Science (SPSS) software (v. 21.0, IBM, Chicago, IL, USA) was used for statistical analysis.

The sample size was calculated for modifications in BCVA and relative retinal sensitivity. For BCVA, based on previous literature, an expected increase of 4 letters with a significance level of 5% and 80% power, assuming a standard deviation of the difference of 5 letters, yielded a required sample size of approximately 25 subjects. For relative retinal sensitivity, a hypothesized modification of 0.5 dB, with the same significance level and power, and assuming a standard deviation of the difference of 1 dB, resulted in a required sample size of approximately 34 subjects. Considering an estimated dropout rate of approximately 15%, the calculated sample size was adjusted to 40 patients. The normal distribution of all quantitative data was verified using the Shapiro-Wilk test; then, parametric or non-parametric tests were used accordingly for comparisons between different time points. The analysis of association between baseline variables and functional outcomes (if there was significant improvement) was performed at 90 days (i.e. BCVA, LLVA, CS) using a linear regression analysis with a stepwise procedure.

All data were presented as mean ± standard deviation. P-values were considered statistically significant if < 0.05.

## Results

We recruited 40 consecutive patients with early or intermediate AMD. Two patients did not complete the PBM protocol: one was lost to follow-up after three PBM applications and was unreachable; the other was hospitalized for a stroke between the screening and baseline visits. Both were excluded from the analysis.

The mean age of the 38 remaining subjects (24 females) was 78.5 ± 6.76 years. Hypercholesterolemia (≥200 mg/dl) was detected in 19 patients (50%) with an overall mean value of 198.68 ± 39,21 mg/dl. Average LDL and HDL levels were 139.22 ± 37.36 mg/dl and 59.46 ± 13.78 mg/dl, respectively. The mean level of triglycerides was 101.33 ± 39.99 mg/dl, with 2 patients having hypertriglyceridemia (≥200 mg/dl). Twenty-four patients (63,16%) were current or former smokers. No patients were taking Age-Related Eye Disease Studies (AREDS) supplements at the beginning of the study or during its duration.

No adverse events were reported during the intervention phase. Two subjects developed exudative macular type 3 neovascularization at W4 and W24 and were promptly treated with anti-VEGF intravitreal injections. These two patients were excluded from the analysis.

### Functional analysis

Baseline mean BCVA (77.82 ± 5.83 ETDRS letters) improved significantly during the six-month follow-up period, peaking at W12 (82.44 ± 5.67 ETDRS letters, *p* < 0.01). At W24, BCVA declined but remained significantly higher than baseline (80.05 ± 5.79 ETDRS letters, *p* < 0.01).

Mean LLVA (61.18 ± 8.58 ETDRS letters at baseline) followed a similar trend, with the highest improvement at W12 (66.33 ± 8.55 ETDRS letters, *p* < 0.01) and a slight decline at W24 (62.05 ± 9.71 ETDRS letters, *p* = 0.02).

Mean CS (17 ± 9.30 ETDRS letters at baseline) improved at W12 (20.11 ± 9.23 ETDRS letters, *p* < 0.01), but returned to baseline levels at W24 (16.45 ± 9.12 ETDRS letters, *p* = 0.5).

For scotopic microperimetry, the mean absolute retinal sensitivity progressively decreased from 9.24 ± 3.44 dB to 7.47 ± 4.41 dB at W24 (*p* < 0.01). Mean relative retinal sensitivity (9.77 ± 3.05 dB) remained stable at W4 (9.21 ± 3.48 dB, p = 0.51) and W12 (9.25 ± 3.68 dB), but decreased at W24 (8.53 ± 3.73 dB, *p* = 0.04). Table 1.

**Table 1:** Functional results of photobiomodulation on early and intermediate age-related macular degeneration patients included in the study.

	Baseline	Week 4	Week 12	Week 24	*P* value*
BCVA (ETDRS Letters)	77.82 ± 5.83	81.24 ± 6.04	82.44 ± 5.67	80.05 ± 5.79	<0.001
LLVA (ETDRS Letters)	61.18 ± 8.58	64.29 ± 11.98	66.33 ± 8.55	62.05 ± 9.71	<0.001
CS (ETDRS Letters)	17 ± 9.30	20.05 ± 9.09	20.11 ± 9.23	16.45 ± 9.12	<0.001
Absolute retinal sensitivity (dB)	9.24 ± 3.44	8.40 ± 4.01	8.28 ± 4.43	7.47 ± 4.41	0.006
Relative retinal sensitivity (dB)	9.77 ± 3.05	9.21 ± 3.48	9.25 ± 3.68	8.53 ± 3.73	0.132

* Related Samples Friedman’s Two-Way Analysis of Variance by Ranks.

*BCVA* best corrected visual acuity, *ETDRS* early treatment diabetic retinopathy study, *LLVA* low luminance visual acuity, *CS* contrast sensitivity.

### Structural analysis

At baseline, HRF were present in 87% of patients, while SDD were present in 53% of patients.

Drusen volume was 0.021 ± 0.011 mm^3^ in the central millimetre; DV3 was 0.165 ± 0.064 mm^3^ and DV6 was 0.493 ± 0.114 mm^3^. DV significantly reduced at W4 (0.018 ± 0.009 mm^3^, *p* < 0.01) and W12 (0.017 ± 0.009 mm^3^, *p* < 0.01); however, at W24 it increased to 0.019 ± 0.012 mm^3^ (*p* = 0.154). DV3 decreased at W4 (0.146 ± 0.056 mm^3^, *p* < 0.01) and W12 (0.143 ± 0.054 mm^3^, *p* < 0.01), but slightly increased at W24, remaining significantly lower than baseline (0.150 ± 0.068 mm^3^, *p* = 0.03). DV6 also decreased from baseline at W4 (0.0459 ± 0.087 mm^3^, *p* < 0.01), W12 (0.0455 ± 0.082 mm^3^, *p* < 0.01), and W24 (0.0464 ± 0.102 mm^3^, *p* < 0.01).

### Post-hoc analysis with fellow eyes

**Fig. 1:**
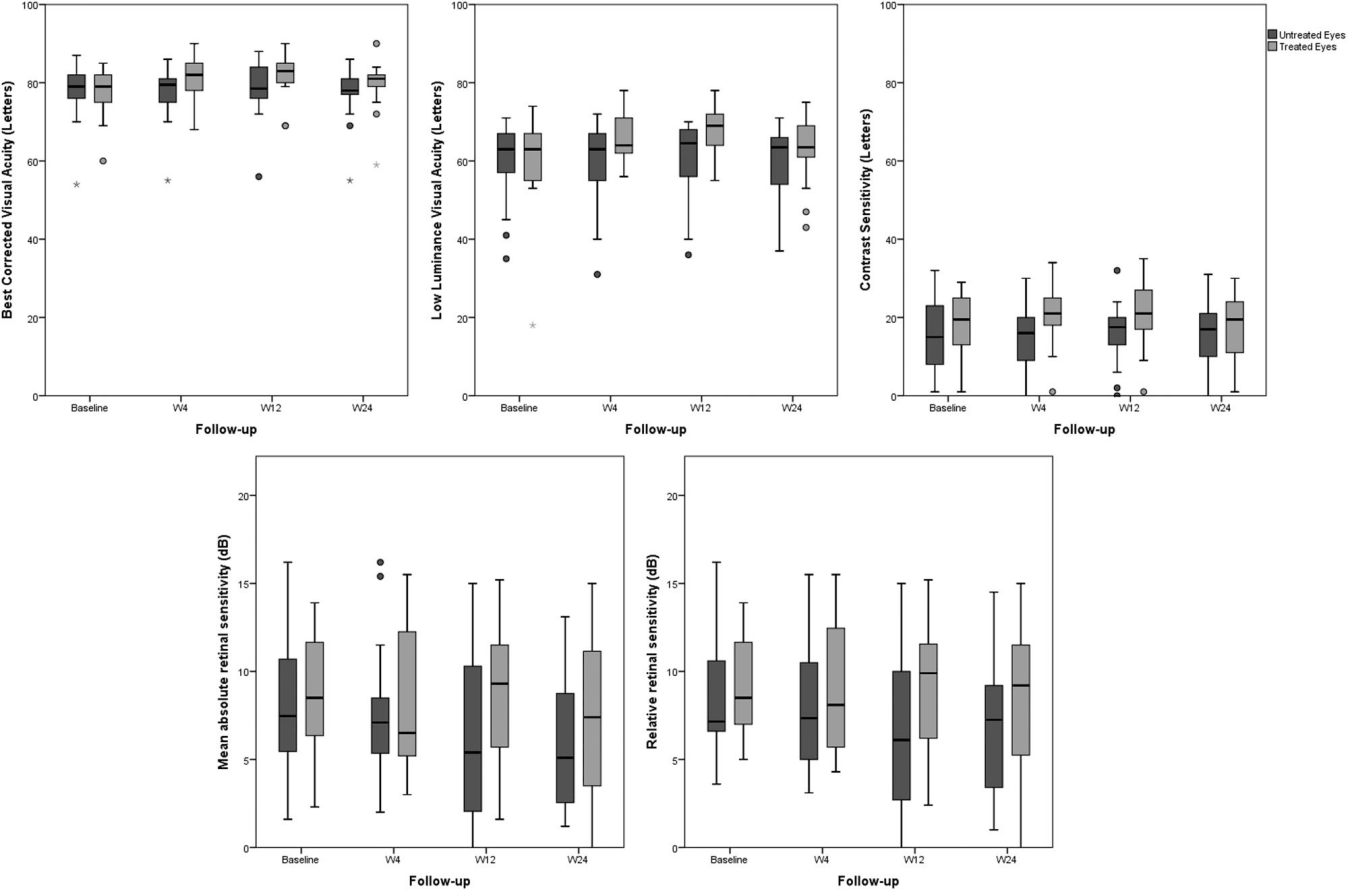
Box-plots showing the post-hoc analysis performed on patients with bilateral early or intermediate age-related macular degeneration. Best corrected visual acuity (top left), low luminance visual acuity (top middle), contrast sensitivity (top right), mean absolute scotopic retinal sensitivity (bottom left) and relative scotopic retinal sensitivity (bottom right) was compared between treated and untreated eyes.

In the subgroup of 18 patients with bilateral early or intermediate AMD, we compared treated and untreated eyes for each functional parameter. Treated eyes showed significant improvement in BCVA (from 77.67 ± 6.32 at baseline, to 82.18 ± 5.97 at W12 [*p* = 0.001] and 79.5 ± 6.44 at W24 [*p* = 0.096]) and, although temporarily, in LLVA (from 60.05 ± 12.49 at baseline, to 68.23 ± 6.78 at W12 [*p* < 0.001] and 63 ± 8.55 at W24 [*p* = 0.104]), and CS (from 18.33 ± 8.34 at baseline, to 21.06 ± 8.58 at W12 [*p* = 0.015] and 17.61 ± 8.84 at W24 [*p* = 0.460]). Untreated eyes showed no changes throughout the follow-up period (Fig. 1). Microperimetric assessment showed no differences between the two eyes. Mean absolute retinal sensitivity significantly decreased between baseline and W24 in both treated (8.8 ± 3.45 dB and 7.44 ± 4.8 dB, respectively, *p* = 0.033) and untreated (8.33 ± 4.01 dB and 6.01 ± 3.79 dB, respectively, *p* = 0.012) eyes (Fig. 1). Mean relative retinal sensitivity remained stable throughout the follow-up in both eyes (Fig. 1).

### Correlations

The difference in BCVA between W12 and baseline (ΔBCVA) was significantly correlated with baseline BCVA (standardized β coefficient = –0.613, *p* < 0.001), baseline CS (β = 0.489, *p* = 0.001) and triglyceride levels (β = –0.371, *p* = 0.006) (Table 2).

**Table 2:** Results of the stepwise linear regression analysis showing the baseline factors that were significantly associated with the improvement of each functional parameter 12 weeks after photobiomodulation.

Dependent variable	Model	Standardized β coefficient	*P* value	R
ΔBCVA	BCVA at Baseline	–0.475	0.004	0.475
	BCVA at Baseline	–0.623	<0.001	0.625
	CS at Baseline	0.433	0.006	
	BCVA at Baseline	–0.613	<0.001	0.724
	CS at Baseline	0.489	0.001	
	Level of Triglycerides	–0.371	0.006	
ΔLLVA	Level of LDL cholesterol	–0.452	0.006	0.452
ΔCS	DV6 at Baseline	0.422	0.012	0.422
	DV6 at Baseline	0.335	0.040	0.524
	LLVA at Baseline	–0.323	0.047	

*BCVA* best corrected visual acuity, *CS* contrast sensitivity, *LLVA* low luminance visual acuity, *LDL* low-density lipoprotein, *DV6* drusen volume in 6 mm.

Improvement in LLVA (ΔLLVA) at W12 was correlated only LDL levels (β = –0.452, *p* = 0.006) (Table 2). Finally, ΔCS at W12 was correlated with DV6 (β = 0.335, *p* = 0.040) and baseline LLVA (β = –0.323, *p* = 0.047) (Table 2).

## Discussion

The objective of this study was to evaluate the effectiveness of PBM in the early and intermediate stages of dry AMD. Previous studies have reported the efficacy of PBM in limited patient populations with dry AMD, with only one being an independent study [17–19]. The PBM4AMD study is among the first independent investigations to introduce PBM in a hospital setting. All enrolled patients successfully completed the entire PBM cycle, which was well tolerated. Only two patients (5.2%) developed MNV during the course of the study (at 1 and 6 months, respectively). These patients were deemed at high risk for developing MNV prior to enrollment (i.e. exudative MNV in the fellow eye and the presence of imaging risk factors [22]). Therefore, these events were considered unrelated to PBM. Previous clinical studies using the same PBM instrument in AMD patients reported an incidence of MNV between 0 and 2.7% [17–19]. However, further studies with larger cohorts are necessary to definitively ascertain any potential association between the therapy and the onset of macular neovascularization.

Functional outcomes, including BCVA, LLVA, and CS, showed statistically significant improvements as early as 1 month after the intervention, with these improvements sustained over the following 2 months. These findings are consistent with previous studies demonstrating the clinical effectiveness of PBM in patients with dry AMD. The observed trend of BCVA improvement at 3 months, which diminishes at 6 months, aligns with previous research, suggesting the temporary nature of PBM’s effects and the potential need for reintervention [16, 17, 19]. Indeed, results from LIGHTSIGHT III demonstrated a sustained 5-letter improvement in BCVA after 1 year of repeated 4-month applications [15].

In a recent independent study, Benlhabib et al. reported a sustained 6-month gain of 5.5 ETDRS letters after a single PBM cycle [18]. The difference with our findings may be due to several factors: their study included only patients with central soft drusen, who might have experienced greater benefits due to drusen resorption over the long term [18] while our study included patients with early and intermediate AMD regardless of central involvement. Additionally, their study employed a different protocol spread over 5 weeks [18]. Further studies are needed to determine whether better patient selection or protocol modifications might enhance functional outcomes.

In addition to BCVA, our study evaluated LLVA and retinal sensitivity under scotopic conditions using microperimetry. LLVA is a good predictor of AMD progression, and scotopic microperimetry assesses rod function, which is primarily affected in this pathology. LLVA significantly increased (+4 ETDRS letters) after 3 months, further demonstrating PBM’s efficacy. However, absolute and relative mean retinal sensitivity under scotopic conditions declined and remained stable, respectively. Our cohort had a 53% prevalence of SDD, which are associated with a profound reduction in scotopic sensitivity [23]. It is plausible that rod function in these patients is already severely compromised, making recovery unlikely.

Drusen volume decreased significantly in all areas at 3 months, with a reduction of 0.022 mm^3^ in the 6 mm ETDRS grid. None of the patients developed geographic atrophy (GA) due to drusen regression, suggesting that PBM may facilitate drusen material reabsorption and potentially slow disease progression. However, drusen growth and collapse are part of the disease’s natural history, possibly making our findings coincidental. Nevertheless, similar findings have been reported in previous PBM studies [17–19] whereas LIGHTSIGHT III found drusen volume remained stable over time, in contrast to growth in the control group [15].

Our study was the first to identify potential biomarkers for predicting functional outcomes. Baseline triglyceride and LDL levels were negative prognostic factors for increases in BCVA and LLVA, respectively. The relationship between blood lipid levels and AMD risk remains debated, with some evidence suggesting a protective role for triglycerides and LDL, while indicating a higher risk associated with HDL [24]. Further validation with larger sample sizes and more comprehensive lipid analyses is needed to draw clinically and prognostically relevant recommendations from our findings. Additionally, clarification of the relationship between lipid biomarkers and PBM efficacy is needed.

Baseline BCVA was inversely correlated with improvement in visual acuity at 3 months, indicating that patients with lower baseline BCVA were more responsive to PBM. However, all included patients had high visual acuity at baseline, which may have influenced this association due to the ceiling effect of current BCVA measuring techniques. The study also found a positive correlation between baseline DV and the increase in CS at 3 months, indicating that patients with higher baseline DV experienced greater reductions and improvements in retinal profile and photoreceptor functionality. The study’s limitations include the absence of a control group, precluding the exclusion of a placebo effect, and the lack of operator blinding. Additionally, the study reported only short-term results, leaving the long-term durability of PBM uncertain. These limitations should be considered when interpreting the results and drawing conclusions and a cautious and thoughtful approach is recommended in the analysis of the collected data. Nonetheless, the study’s strengths include its prospective design and comprehensive multimodal evaluation of patients in a specialized retinal pathology center.

In conclusion, this independent prospective study conducted in a hospital setting evaluated PBM for early and intermediate AMD. We observed temporary functional improvements and identified potential biomarkers associated with therapy outcomes. It is important to note that although statistically significant, the magnitude of functional improvement may not be clinically significant, especially for patients with already high visual acuity who may not perceive a qualitative difference. This also applies to the reduction of drusen volume, which still poses a risk for GA induction, not entirely ruled out in a short-term study.

The primary goal of treatment in early or intermediate AMD is to reduce the risk of developing late AMD. Based on the present results, PBM cannot be unequivocally interpreted as a treatment but rather as a potential temporary stabilization of the disease. In the context of stabilization, it may be necessary to perform multiple treatments to maintain the benefits provided. However, we still do not know the long-term safety implications of repeated applications, as there are currently no data extending beyond two years. PBM is registered as a medical device and is already available for application in private practices in several European countries; however, long-term efficacy and safety outcomes are still limited. Further independent studies are necessary to validate the current findings and to optimize patient selection for PBM.

## Summary

### What was known before:

- Photobiomodulation (PBM) utilizes specific wavelengths of light, particularly near-infrared light, to modulate cellular pathways. Current hypotheses speculate that it promotes increased mitochondrial activity and cellular metabolism.
- Age-related macular degeneration (AMD) is a chronic retinal disease causing irreversible central vision loss. Current treatments target advanced stages, but PBM, explored in clinical trials for early and intermediate AMD, shows promising safety and visual outcome results, with efficacy and prognostic factors still under investigation.

### What this study adds:

- The study demonstrated significant improvements in functional and structural outcomes, albeit temporary and of small magnitude.
- The findings from this study provide valuable insights into the potential of PBM to stabilise early and intermediate stages of dry AMD.
- The study’s outcomes underscore the importance of further research to validate and optimize patient selection for PBM therapy in AMD.

## Data Availability

Data supporting this study are available upon reasonable request to the corresponding author.
